# Deep-Ultraviolet Photodetectors Based on Epitaxial ZnGa_2_O_4_ Thin Films

**DOI:** 10.1038/s41598-018-32412-3

**Published:** 2018-09-19

**Authors:** Si-Han Tsai, Sarbani Basu, Chiung-Yi Huang, Liang-Ching Hsu, Yan-Gu Lin, Ray-Hua Horng

**Affiliations:** 1 Institute of Electronics, National Chiao Tung University, Hsinchu, 300 Taiwan Republic of China; 2 National Synchrotron Radiation Research Center (NSRRC), Hsinchu, 300 Taiwan Republic of China; 3 Center for Emergent Functional Matter Science, National Chiao Tung University, Hsinchu, 300 Taiwan Republic of China

## Abstract

A single-crystalline ZnGa_2_O_4_ epilayer was successfully grown on c-plane (0001) sapphire substrate by metal-organic chemical vapor deposition. This epilayer was used as a ternary oxide semiconductor for application in high-performance metal–semiconductor–metal photoconductive deep-ultraviolet (DUV) photodetectors (PDs). At a bias of 5 V, the annealed ZnGa_2_O_4_ PDs showed better performance with a considerably low dark current of 1 pA, a responsivity of 86.3 A/W, cut-off wavelength of 280 nm, and a high DUV-to-visible discrimination ratio of approximately 10^7^ upon exposure to 230 nm DUV illumination than that of as-grown ZnGa_2_O_4_ PDs. The as-grown PDs presented a dark current of 0.5 mA, a responsivity of 2782 A/W at 230 nm, and a photo-to-dark current contrast ratio of approximately one order. The rise time of annealed PDs was 0.5 s, and the relatively quick decay time was 0.7 s. The present results demonstrate that annealing process can reduce the oxygen vacancy defects and be potentially applied in ZnGa_2_O_4_ film-based DUV PD devices, which have been rarely reported in previous studies.

## Introduction

Recently, deep-ultraviolet (DUV, 190–350 nm) photodetectors (PDs) have attracted much attention due to their broad applications in the civil and military fields, such as engine control, solar UV monitoring, astronomy, lithography aligners, secure space-to-space communications, or missile detection^1–4^. These UV instruments are always operated at high temperature and harsh environments. Thus, several materials have been introduced to fabricate PDs. High photoresponsivity, low noise levels, high spectral selectivity, and high stability are required for PDs in such applications. Over the past decades, several semiconductor solar-blind PDs have obtained considerable achievements. A variety of well-established Si-based UV PDs are available in the market. However, UV-enhanced Si-based PDs exhibit limitations due to their low bandgap and requirement of expensive optical filters for spectral selectivity. Moreover, Si-based PDs efficiency can be degraded with increased temperature. To solve this problem, wide bandgap semiconductor materials, such as Group III nitrides: GaN, (Eg, ~3.4 eV) and AlN (~6.2 eV) and their ternary compounds, namely, Al_x_Ga_1-x_N (x > 40% with 6 eV) and SiC have been widely used for solar-blind and visible-blind UV PDs^5,6^. Nevertheless, high-density defects, such as dislocations and grain boundaries, can be generated during epitaxial growth of AlGaN films on sapphire substrate because of the lattice mismatch. On the other hand, persistent photoconductivity (PPC) effect also occurs when the free carrier density is equal to the trap density. Therefore, the most challenging aspects are to attain low dark current and high responsivity for the application of DUV PDs. Feng *et al*.^7^ reported the first individual β-Ga_2_O_3_ nanowires as solar-blind PDs upon exposure to 254 nm light illumination; these nanowires present upper limits and response and recovery times of 0.22 and 0.09 s, respectively. Our previous studies also demonstrated the effects of crystallinity and point defects on the time-dependent photoresponsivity and cathodoluminescence (CL) properties of β-Ga_2_O_3_ grown by metalorganic chemical vapor deposition (MOCVD)^8^. PDs fabricated using 700 °C annealed β-Ga_2_O_3_ epilayers produced substantially low dark current of ~4 pA and large photocurrent-to-dark current contrast ratio of ~10^5^ ^8^. It is worthy to mention that ternary metal oxides have attracted significant attention due to its large bandgap and high wavelength selectivity. However, there is insufficient study regarding ternary metal oxide alloy PDs. Recently, Li *et al*. demonstrated an improved DUV-to-visible ratio of about 10^4^, relatively fast response speed, low dark current of <0.1 pA, and a responsivity of 38.3 A/W (gain of ~200) for individual Zn_2_GeO_4_ nanowire visible-blind DUV PDs^9^. Our previous studies have also shown the high feasibility of single-crystalline spinel-shaped ZnGa_2_O_4_ films prepared by MOCVD. These films have been fabricated to metal–oxide–semiconductor field-effect transistors^10^. The correlations among crystal, microstructural, morphological, compositional, CL, and electrical properties of these ZnGa_2_O_4_ films were also analyzed in details in our previous study^11^. The calculated carrier concentration, mobility, and resistivity of the ZnGa_2_O_4_ films were 6.72 × 10^16^ cm^−3^, 1.4 cm^2^/Vs, and 67.9 Ω-cm, respectively. These values indicate that ZnGa_2_O_4_ films are promising for several optoelectronic applications^12^. In this work, the solar-blind PDs made of the ZnGa_2_O_4_ epilayers grown by MOCVD were studied. To further understand the annealing effect on the DUV PDs performance, as-grown and annealed PDs were also fabricated and their properties were studied in details. The corresponding crystalline, optical, and electrical properties of the ZnGa_2_O_4_ epilayers before and after thermal annealing were measured by X-ray diffraction (XRD) and X-ray photoelectron spectroscopy (XPS). The above properties related to the performance of DUV PDs were discussed in this paper.

## Results and Discussion

**Figure 1:**
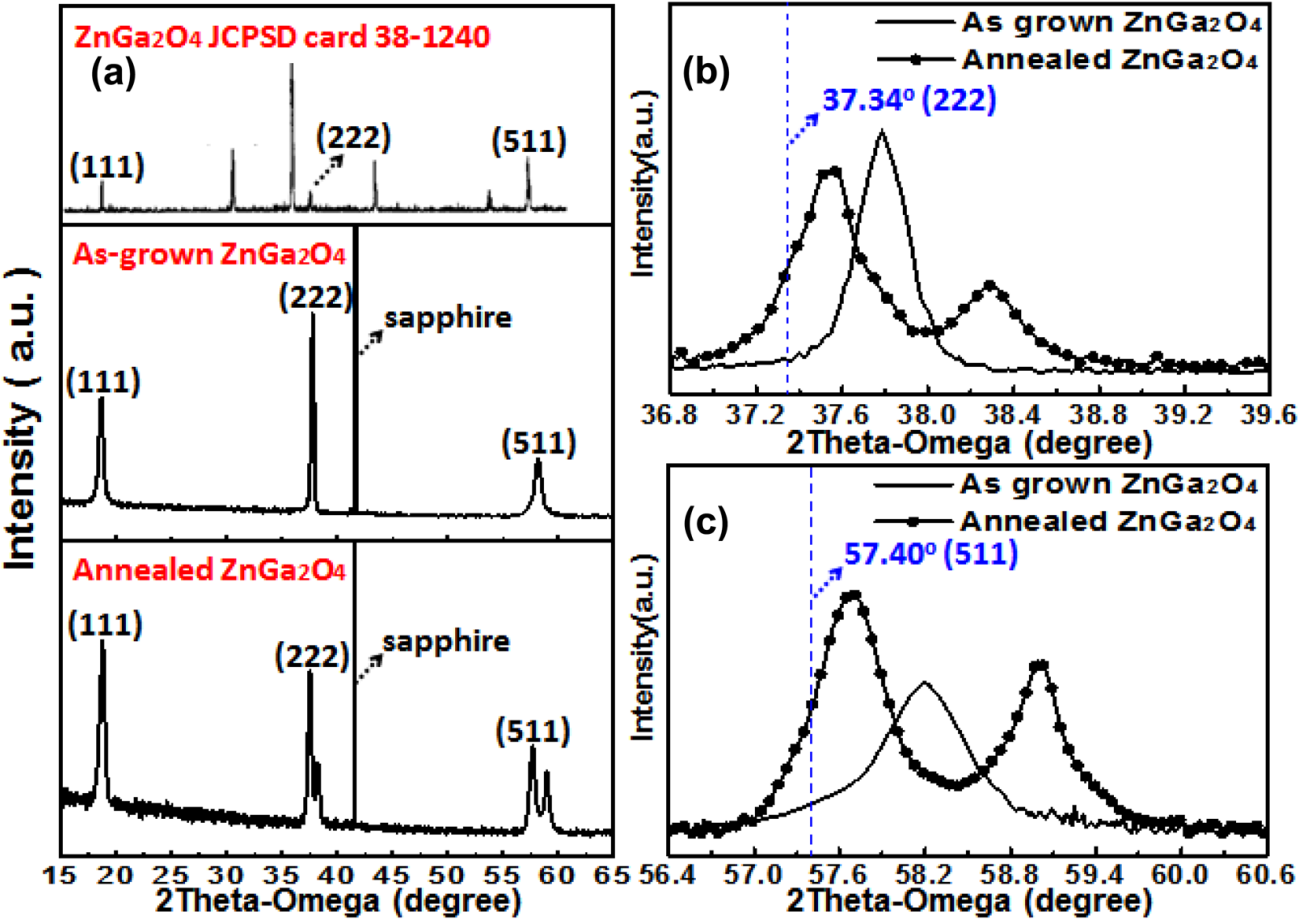
(**a**) Standard X-ray diffraction spectra of ternary compound ZnGa_2_O_4_ and the as-grown and annealed ZnGa_2_O_4_ thin films. (**b**) Diffraction peaks corresponding to the diffraction plane (111) of as-grown and annealed ZnGa_2_O_4_ thin films. (**c**) Diffraction peak corresponding to the diffraction plane (222) of as-grown and annealed ZnGa_2_O_4_ thin films.

The crystallinity of ZnGa_2_O_4_ films with and without thermal annealing was measured by XRD, as shown in Fig. 1(a). The crystal orientations of as-grown ZnGa_2_O_4_ films were characterized with evident XRD peaks at 18.67°, 37.77°, and 58.17°, which can be specified to (111), (222), and (511) diffraction planes. After furnace annealing treatment, the XRD peaks of ZnGa_2_O_4_ presented shifting phenomena. For comparison, the peaks of 18.40°, 37.34°, and 57.40° of standard ZnGa_2_O_4_ (JCPDS card 38–1240), which corresponded to (111), (222), and (511) diffraction planes of the cubic spinel crystal structure^13^ was also shown in Fig. 1(a). In order to analysis more clearly, the peaks corresponding to the (222) and (511) diffraction planes were magnified and shown in Fig. 1(b,c). It was found that the XRD peaks corresponding to (222) diffraction plane for annealed ZnGa_2_O_4_ shifted from 37.77° to 37.55°. Similarly, the XRD peaks corresponding to (511) diffraction plane for annealed ZnGa_2_O_4_ shifted from 58.17° to 57.65°. These results indicated that the ZnGa_2_O_4_ film annealed in nitrogen ambient is close to the ZnGa_2_O_4_ cubic spinel structure. Moreover, the XRD peaks at approximately 38.25° and 59.01°, which were not observed in as-grown ZnGa_2_O_4_ film, were obtained in the annealed ZnGa_2_O_4_ thin film. They are the diffraction peaks of β-Ga_2_O_3_ monoclinic crystal structure. It means that the ($\bar{4}02)$ and ($\bar{6}03$) diffraction planes for the standard β-Ga_2_O_3_ monoclinic crystal structure appeared in annealed film. This suggests that the recrystallization phenomenon made gallium and oxygen reorganize the β-Ga_2_O_3_ crystal through annealing process.

**Figure 2:**
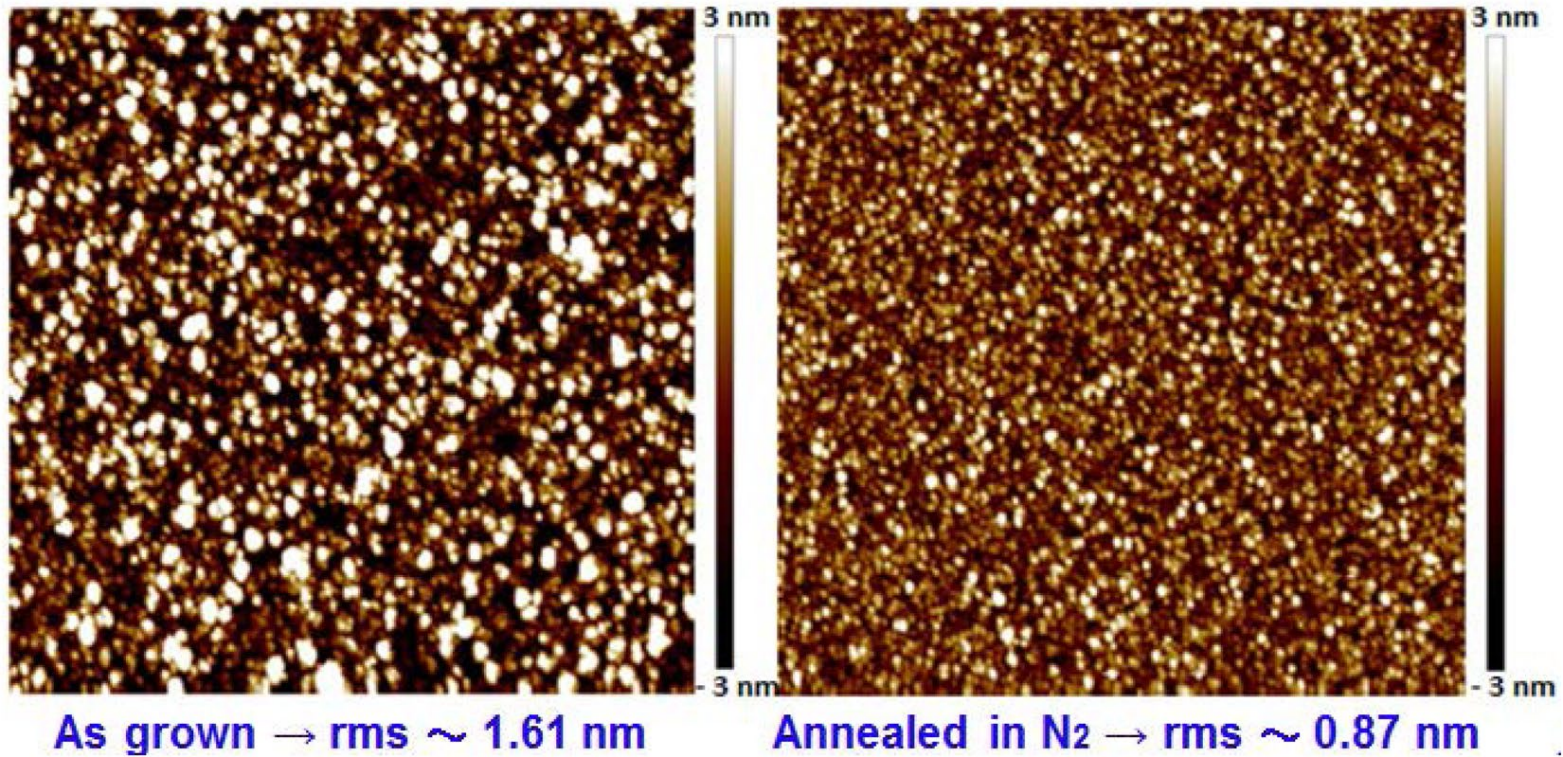
Atomic force microscope images of thin-film surfaces and surface roughness of as-grown and annealed ZnGa_2_O_4_ thin films.

To further investigate the annealing effect in the ZnGa_2_O_4_ film, the surface morphologies (scanning length of 5 µm) of typical as-grown and annealed ZnGa_2_O_4_ films on sapphire were examined. The root-mean-square roughness values for the ZnGa_2_O_4_ films with and without annealing were 1.61 and 0.87 nm, respectively, as shown in Fig. 2(a,b). The annealed ZnGa_2_O_4_ film displayed relatively low surface roughness. The particle/agglomerate distribution was more uniform than that of the as-grown films. Therefore, annealing treatment resulted in smooth film surface. It is well known that the defects (e.g. oxygen vacancies and dangling bonds) always exist in the bulk and the surface of the epilayers. They were responsible in trapping the photogenerated electron or hole carriers. The smooth surface would lead to the low surface area and then the low surface density states for the annealed ZnGa_2_O_4_ film, which is beneficial for the improved performance of the PD^14,15^.

**Figure 3:**
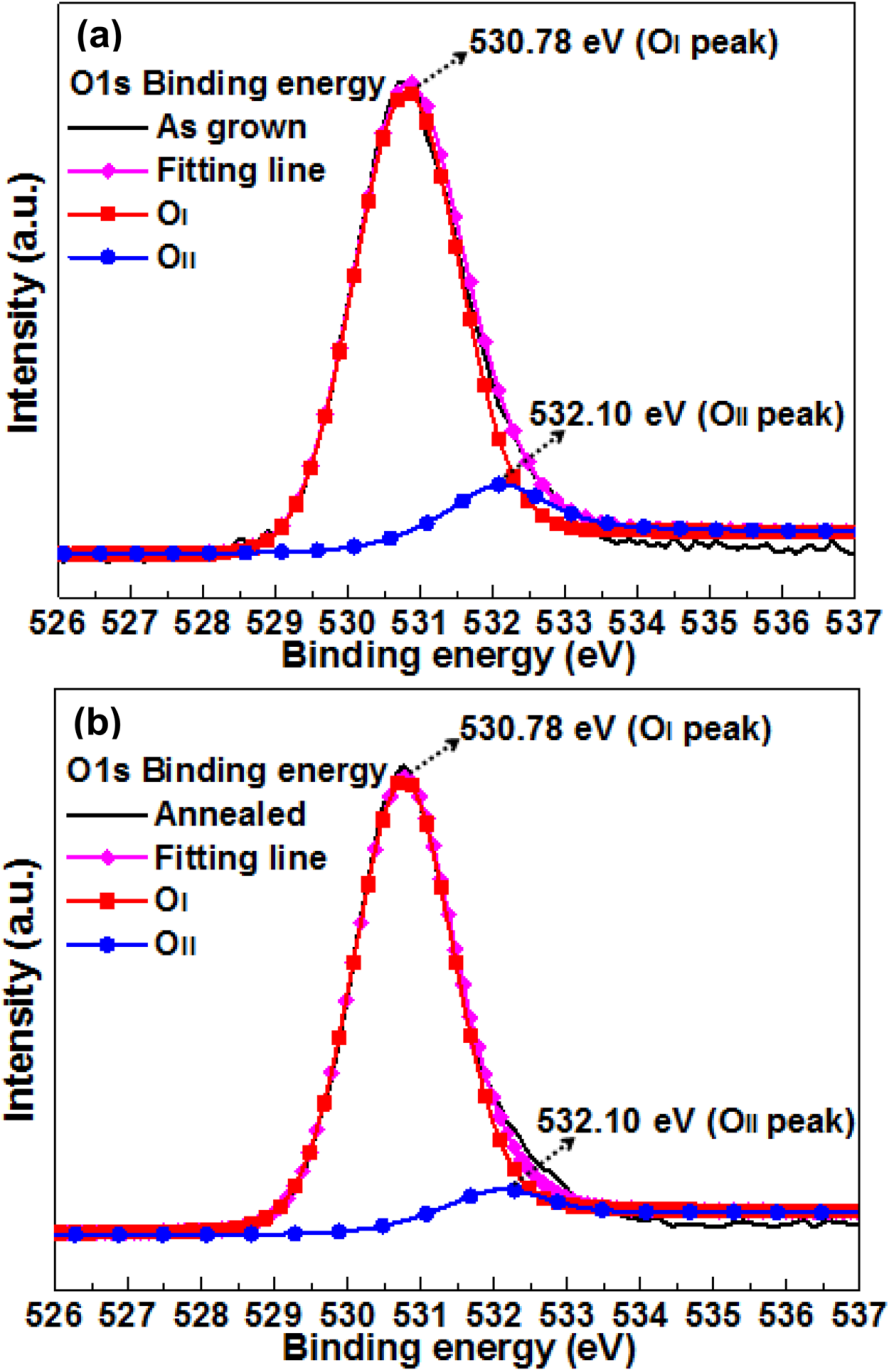
X-ray photoelectron spectroscopy spectra of O1s core level for (**a**) as-grown and (**b**) annealed ZnGa_2_O_4_.

XPS analysis can be used to characterize the surface chemical composition and bonding states of as-grown and annealed ZnGa_2_O_4_ films quantitatively. Figure 3(a,b) show the O1s core-level XPS spectra of as-grown and annealed ZnGa_2_O_4_ films, respectively. These XPS spectra were all fitted well with Gaussian functions. Figure 3(a,b) illustrate that the O1s core-level peaks of ZnGa_2_O_4_ (as-grown and annealed samples) can be divided into two components, namely, I and II, which were positioned at 530.78 and 532.10 eV, respectively. Peak I is assigned to lattice oxygen ions, and peak II is designated to the oxygen ions in the oxygen vacancies region^16^. Notably, the oxygen vacancy in an oxygen-deficient interlayer acted as an electron trap that can control the conductivity of oxide semiconductors^17^. Consequently, the oxygen vacancy proportion can correspond to conductive basis. The calculated area covered by the peak ratio of II/I was reduced from 0.167 to 0.11 after thermal annealing treatment. This result is consistent with the decrease in photocurrent after the annealing treatment^18^.

**Figure 4:**
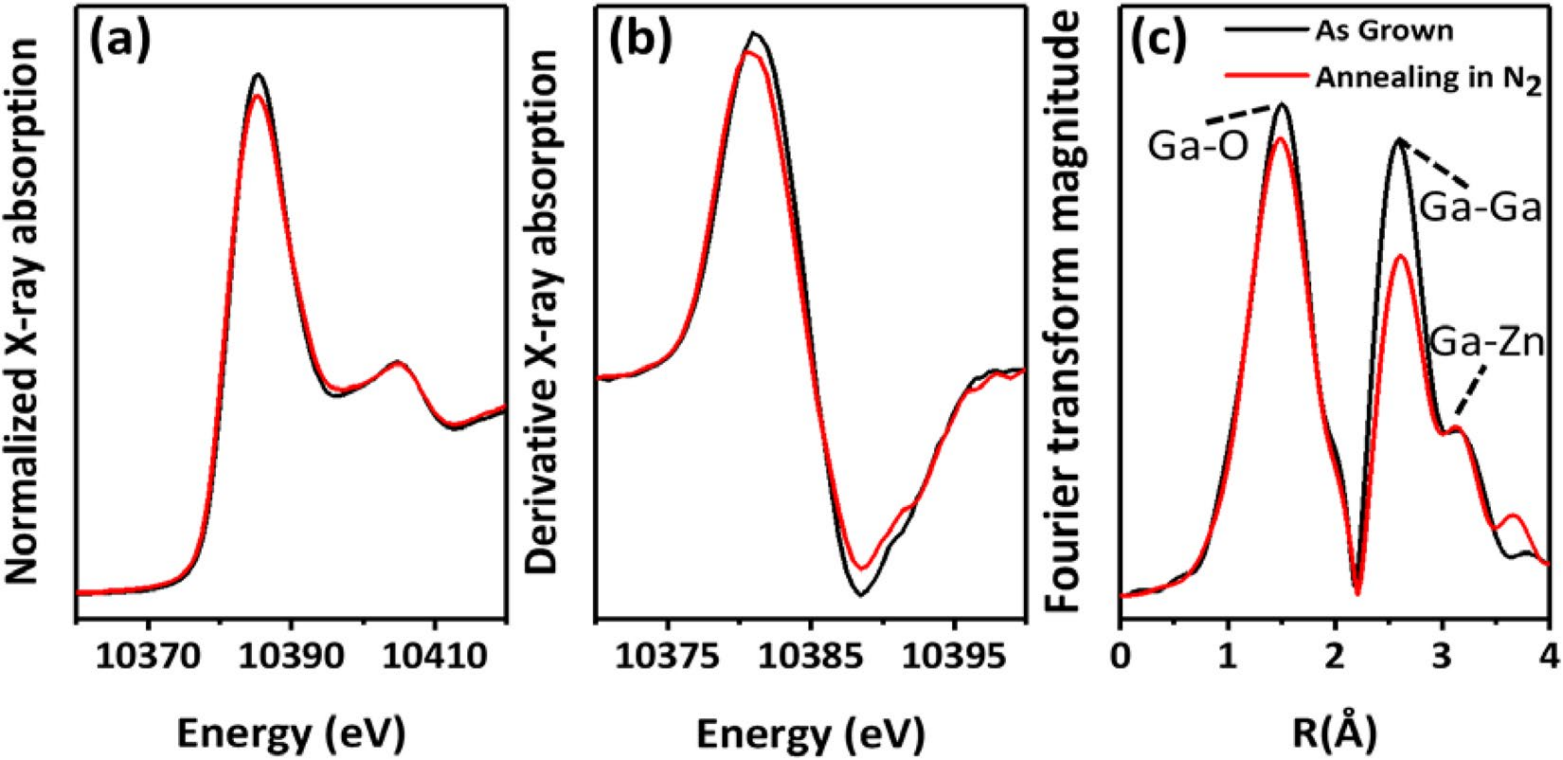
Ga K-edge (**a**) XANES, (**b**) derivative XANES, and (**c**) EXAFS spectra of ZnGa_2_O_4_.

The XANES shape of Ga K-edge spectra, which were dominated with geometry around Ga atoms, is shown in Fig. 4(a). The white-line feature and side hump are fitted to ZnGa_2_O_4_^19^. The adsorption edges of as-grown and annealed ZnGa_2_O_4_ were located at 10381.2 and 10380.7 eV, respectively. The white-line intensity of annealed sample was slightly lower than that of the as-grown one. The change and shifted energies of white-line feature can be attributed mainly to the disordered and different coordination environments^20^. In addition, the negative shift of the adsorption edge occurs with decreased coordination, which can be used as an indicator of the coordination number of O atom in the structure^21^. The Fourier transform EXAFS spectra of Ga K-edge were used to further understand the atomic structure of ZnGa_2_O_4_ film. Figure 4(b) shows the magnitude of the Fourier transforms of EXAFS functions. The first shell of Ga–O bonding (1–2 Å) slightly decreased after annealing treatment, which indicated the decreased coordination of O atoms. This phenomenon can be attributed to the decreased GaO_6_ octahedron and GaO_3_ tetrahedron ratio, which showed a trend similar to that of XANES results. Moreover, the annealed sample amplitudes showed a progressive decrease in the second shell due to the Ga–Ga bonding at 2–2.5 Å. By contrast, the Ga–Zn bonding amplitude (3–3.5 Å) was unaffected with the annealing treatment. This decrease in Ga–Ga amplitudes was caused by the disordered structure or phase transition between ZnGa_2_O_4_ and Ga_2_O_3_ as shown in Fig. 4(c). XAS results indicated that as-grown ZnGa_2_O_4_ was dominated with ZnGa_2_O_4_, and part of ZnGa_2_O_4_ structure may transform into Ga_2_O_3_ after annealing treatment. The obtained results well agreed with the observation found using XRD.

**Figure 5:**
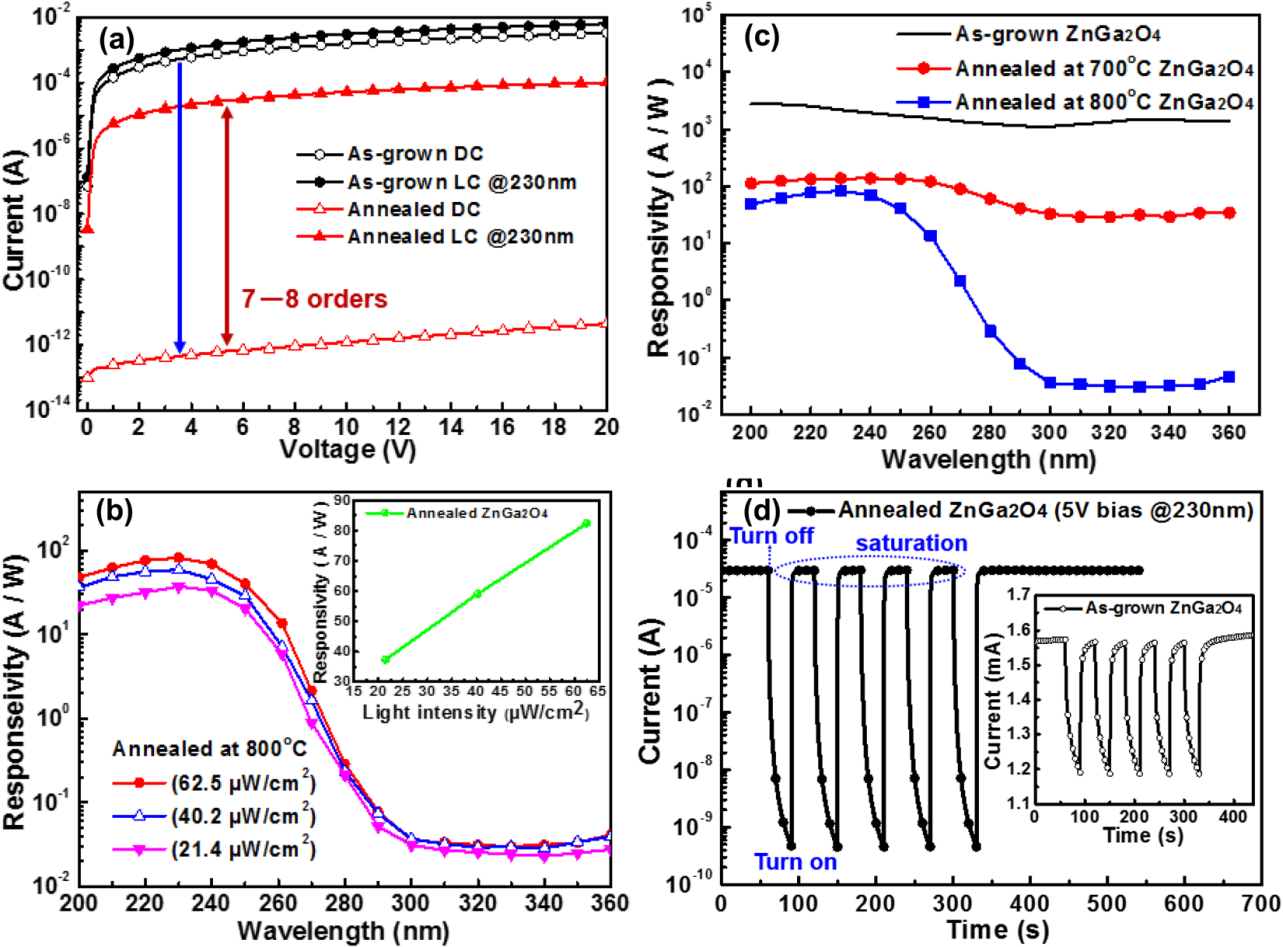
(**a**) Dark current (DC) curve and photocurrent (LC) at 230 nm curve for as-grown (black) and annealed in 800 °C (red) ZnGa_2_O_4_ UPDs. (**b**) Responsivity spectra of UV region for as-grown (black), annealed in 700 °C (red) and 800 °C (blue) ZnGa_2_O_4_ UPDs and the inset is the responsivity as a function of light intensity. (**c**) Spectral responsivities of MSM devices with varying light intensities (~21.4, 40.2 and 62.5 μW/cm^2^) of the N_2_-annealed (800 °C) ZnGa_2_O_4_ UPDs at a 5 V bias. We have plotted Normalized responsivity (in logarithmic scale) to compare the out of band rejection ratio of the devices. The spectral responsivity measurement done by using a standard UV-enhanced Si Photodiode. After annealing, the solar blind selectivity of the ZnGa_2_O_4_ by 3 to 4 orders as compare to as-grown devices. (**d**) Dynamic photoresponse at 230 nm with 5 V bias of annealed (800 °C) ZnGa_2_O_4_ DUV PDs and the inset is the corresponding dynamic photoresponse of as-grown ZnGa_2_O_4_ DUV PDs.

In this study, ZnGa_2_O_4_ epilayers displayed the wide bandgap (Eg, ~5.2 eV) responses to UV-C band (200–290 nm) and solar blindness to UV-A/B (290–400 nm). The bandgap measured by CL for the as-grown and annealed ZnGa_2_O_4_ epilayers have been discussed in the Supporting Text (Supplementary Information 1). Therefore, the ZnGa_2_O_4_ PD performance should be evaluated. I–V curves with and without UV illumination (DUV, 230 nm; power density, ~62.5 μW/cm^2^) for PDs based on the as-grown and annealed ZnGa_2_O_4_ films are displayed in Fig. 5(a). The photocurrent increased with UV illumination or applied bias voltage for both devices at forward bias voltage due to the increased carrier drift velocity. This observation suggested that the films possessed photoconductive properties. Annealed ZnGa_2_O_4_ films presented a much lower dark current (Idark) at a bias of 20 V than that of the as-deposited films. The low dark current was attributed to the low concentration of the oxygen vacancies or low carrier density in the ZnGa_2_O_4_ films. Post-annealing affected the crystalline quality, surface properties, altered defects, and electrical properties of ZnGa_2_O_4_ films. Our previous studies have demonstrated the quality of as-grown ZnGa_2_O_4_ films, which contain lower degree of crystallinity and higher point defects than those of annealed ZnGa_2_O_4_ films^11^. The dark current of as-grown ZnGa_2_O_4_ film PDs (Idark ~1 mA at a 20 V bias) was approximately 10^8^ times higher than that of annealed PDs based on ZnGa_2_O_4_ films so that the photocurrent stimulated by DUV light was difficult to distinguish. High Idark for as-grown ZnGa_2_O_4_ films can be inter-related to the high intrinsic defects, such as oxygen vacancies and/or surface density state in the ZnGa_2_O_4_ films. The low photocurrent implied that the defect concentration was reduced, which resulted in low conductivity^15^. Surface-related defects can act as adsorption sites or trapping center to capture free electrons in ZnGa_2_O_4_ films. These trapping centers could result in current leakage. Therefore, the surface states enhance the current leakage and nonradiative recombination. Annealed ZnGa_2_O_4_ film-based detectors also showed low current leakage, which was below 1 pA, at 5 V reverse bias compared with those of as-grown counterparts due to the improved quality of material. Under a 230 nm (62.5 μW/cm^2^) DUV illumination, the current increased by approximately one order of magnitude compared with that of the dark current for as-grown ZnGa_2_O_4_ PDs. However, the PDs made of annealed samples exhibited much higher photocurrent-to-dark current ratio of ~10^8^, which is better than that of the reported MSM photodiodes^22–24^.

Responsivity (R) is a significant detector parameter, which is defined as the ratio of the number of incident photons per incident optical power: R = I_ph_/P_inc_ = (I_light_ - I_dark_)/P_inc_, where P_inc_ is the incident optical power in watts, I_ph_ is the photocurrent. The plot of responsivity as a function of wavelength provided the spectral response of the detector. The PD responsivities with varying light intensities (~21.4, 40.2 and 62.5 μW/cm^2^) as a function of wavelength at an applied bias of 5 V for the ZnGa_2_O_4_ films annealed at 800 °C were investigated and shown in Fig. 5(b). Due to the light source limitations, the responsivity at a higher light intensity more than 62.5 μW/cm^2^ cannot be measured. The inset in Fig. 5(b) shows the responsivity versus intensity curve maintained almost linear.

In order to investigate the influence of thermal annealing on the performance of ZnGa_2_O_4_ based solar blind photodetectors, different DUV PDs were fabricated, where ZnGa_2_O_4_ epilayers were thermal annealed at 700, 800 and 900 °C in nitrogen ambient for 1 hour. The spectral responsivity of the annealed DUV PDs was measured and compared with those of as-grown ZnGa_2_O_4_ based PDs as illustrates in Fig. 5(c). It can be clearly observed that the variation of photoresponse properties of DUV PDs could be attributed to oxygen vacancies effects during the different temperature annealing process. For the ohmic-type PDs based on the as-grown and annealed at 700 °C, ZnGa_2_O_4_ epitaxial films presented no selectivity of responsivity in DUV region. On the contrary, the device based on the 800 °C annealing had a high rejection ratio of 104 from 240 nm to 320 nm band, as shown in Fig. 5(c). Furthermore, it decreases to 103 (not shown here) for 900 °C annealed ZnGa_2_O_4_ PDs devices. Thus 800 °C is the optimized annealing temperature used to fabricate the DUV PDs, which exhibited strong selectivity to solar blind radiation without interference with UV-A (320–400 nm) and visible radiation. The measured absolute peak responsivity for as-grown, 700 °C, 800 °C, and 900 °C annealed devices were 2782, 136.3, 86.3 and 0.007 A/W at 230 nm, respectively. Therefore, the PDs made of ZnGa_2_O_4_ epilayers annealed 900 °C can not be used as DUV PDs due to low responsivity. It was worthy to mention that the responsivity for the annealed ZnGa_2_O_4_ PDs devices dropped significantly (Fig. 5(c)), which is consistent to those reported in earlier studies^12,22^. Even though, the selectivity of the DUV PDs made of ZnGa_2_O_4_ annealed at 800 °C presents the highest for these DUV PDs. The detailed dark current (Idark) and UV-illuminated photocurrent (Ilight) at 230 nm I–V curves for MSM UPDs fabricated from as-grown and annealed (700, 800, 900 °C) ZnGa_2_O_4_ epitaxial films have been discussed in the Supporting Text (Supplementary information 2).

Moreover, no response was given to the UV-A/B, and almost 90% of peak intensity was obtained as the wavelength shorter than 280 nm for PDs made of 800 °C annealed ZnGa_2_O_4_. Therefore, the sharp cutoff and obviously high rejection power of this PD proved its potential as a candidate for solar-blind DUV detection application. Although the DUV PD made of as-grown ZnGa_2_O_4_ films presented such high responsivity of ~2782 A/W (under 230 nm DUV), the selectivity to spectral responsivity was too low to use. As-grown ZnGa_2_O_4_ films contained higher defects than those of annealed ZnGa_2_O_4_ films. The high responsivity was due to the defects in the as-grown films, which resulted in trapping states that can enhance internal gain. Zheng *et al*.^25^ also demonstrated that the high responsivity of Mg_0.46_Zn_0.54_O film-based detectors is attributed to the long life time of photo-ionized holes trapped in the deep level, as precisely measured using deep-level transient spectral technique. The long life time is commonly induced by the trap states of holes, which include the interface states between semiconductor and electrode, surface states, and deep level defects^14^. After furnace annealing, the responsivity of UVA region (310–400 nm) decreased by about three orders of magnitude for ZnGa_2_O_4_ film detectors. The spectral response of annealed ZnGa_2_O_4_-based detectors was in the range of 210–240 nm.

Response time is another important parameter to determine the suitability of detector for specific application. The response time is a measurement of the time required for photo signal to increase or decay to a specific value and defined as the time to reach 90% saturation response. Decay time is the time taken by the PDs to decay 10% of the maximum photocurrent when the UV light is switched off. The transient continuous photoresponse behavior of the as-grown and annealed ZnGa_2_O_4_ PDs was investigated under 230 nm DUV light with an intensity of ~62.5 µW/cm^2^ and at a bias of 5 V. PDs were measured by periodically turning on and off the UV light. The rise and decay time at 5 V bias were more than 10 s for the as-grown ZnGa_2_O_4_ PDs, shown in the inset of Fig. 5(d). Correspondently, the measured typical rise and decay time are 0.5 and 0.7 s, respectively, for the DUV PDs fabricated using annealed ZnGa_2_O_4_ films as shown in Fig. 5(d). Obviously, there exists the PPC effect in the as-grown ZnGa_2_O_4_ PDs. The PPC effect is a light-induced enhancement in the conductivity that persists for a long period after the termination of light excitation. It was resulted from the deep level traps. In the as-grown ZnGa_2_O_4_, the deep level traps comes from the oxygen vacancy and surface density states which could be demonstrated by CL, AFM and I-V characteristics. These results agreed well with recently published results about ZnGa_2_O_4_-based PDs^23,24,26^. On the other hand, there was no PPC effect and no noticeable change in photocurrent upon turning on and off the light illumination over multicycles for the annealed ZnGa_2_O_4_ PDs. This continuous variation in photocurrent of annealed ZnGa_2_O_4_ film PDs indicated excellent reproducibility and stability. Evidently, the annealing treatment alleviates the PPC effect^27^. Additionally, the complete response of as-grown and annealed (800 °C) ZnGa_2_O_4_ DUV PDs are presented in Fig. S3 (Supplementary Information 3).

**Figure 6:**
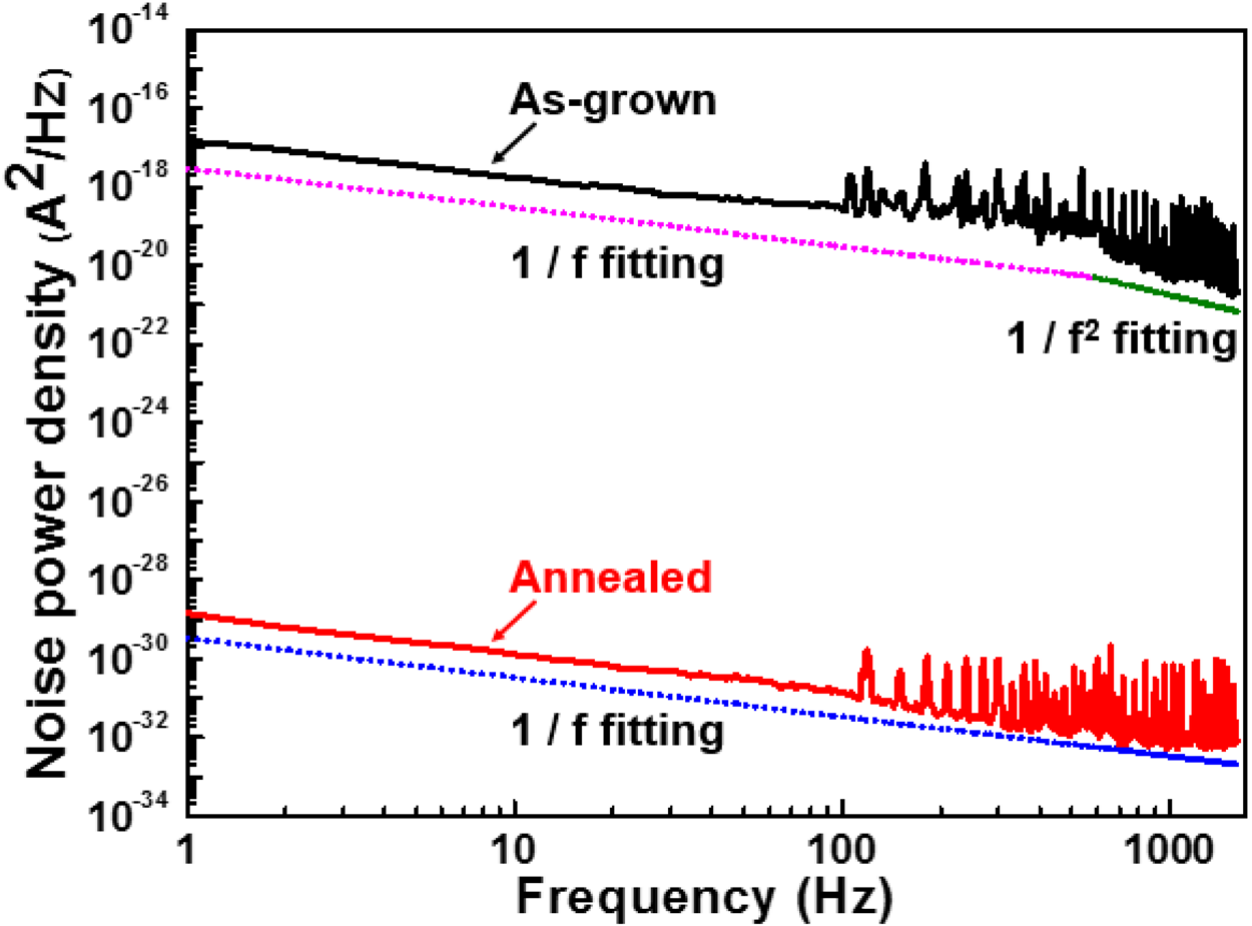
Noise power density of as grown and annealed ZnGa_2_O_4_ UPDs.

Figure 6 displays the noise power density of ZnGa_2_O_4_ thin film with and without annealing at 5 V bias. The frequency range was tuned from 1 Hz to 1600 Hz. The obtained spectra from the PD can be fitted well by the Hooge-type equation as follows^28^.

$${{S}}_{{n}}({f})={{S}}_{{o}}(\frac{{{I}}_{{d}}^{{\beta }}}{{{f}}^{{\alpha }}}),$$

where *I*_*d*_ is the dark current, *f* is the frequency, S_o_ is a constant, and α and β are two fitting parameters. The fitting curve of noise power density spectra for two ZnGa_2_O_4_ DUV PDs (Fig. 6) was similar to 1/*f* with the frequency at < 500 Hz. The pure 1/*f* noise indicated that the noises at <500 Hz were related to flicker noise. Flicker noise or 1/*f* -type noise mainly originates from the acoustic phonons and ionized impurities that cause mobility fluctuation through lattice and impurity scatterings^29^. The noises at >500 Hz for as-grown ZnGa_2_O_4_ DUV PD decreased as 1/*f*^2^ due to generation-recombination noise. Obviously, generation-recombination noise can be reduced by thermal annealing due to the oxygen vacancy reducing. With these values, we can determine the constant S_o_ from Fig. 6. The So values for as-grown and annealed ZnGa_2_O_4_ DUV PDs were 1.70 × 10^−7^ and 1.3 × 10^−7^, respectively. At a bandwidth (B) of 1000 Hz, the total square noise current <in^2^ > can be estimated by integrating S_n_*(f)*.

In general, we assume that S_n_*(f)* = S_n_(1 Hz) when *f* < 1 Hz. Thus, the noise equivalent power (NEP) and detectivity (D*) are given as follows.

$$\begin{array}{c}{\langle {in}\rangle }^{2}={\int }_{0}^{{B}}{{S}}_{{n}}({f})df={\int }_{0}^{{\rm{1}}}{{S}}_{{n}}({\rm{1}})df+{\int }_{1}^{{B}}{{S}}_{{n}}({f})df={S}_{{o}}[{\rm{In}}({B})+1],\\ {NEP}=\frac{\sqrt{{\langle {in}\rangle }^{2}}}{{R}}\,{{D}}^{\ast }=\frac{\sqrt{{A}}\sqrt{{B}}}{{NEP}},\end{array}$$

where *R* is the responsivity of the DUV PDs, and A and B are the active areas of the PD and the bandwidth. The NEP of as-grown and annealed ZnGa_2_O_4_ PDs was approximately 9.33 × 10^−9^ and 9.994 × 10^−12^ W, which corresponded to D* of 2.449 × 10^8^ and 2.2870 × 10^11^ cm Hz ^0.5^ W^−1^, respectively.

Moreover, Table S1 (Supplementary Information 4) shows the performance comparison with recently published ternary oxide-based PDs. Inadequate studies have been conducted on these types of PDs. The ZnGa_2_O_4_ thin-film PDs presented the potential to achieve the highest UV-to-visible ratio in the order of 10^7^, lowest dark current of 1 pA, and short response time compared with those of other PDs based on MSM structure^30–36^.

This result suggested that thermal annealing treatment increased the PD detectivity. Furthermore, the detectivity of annealed ZnGa_2_O_4_ thin film was suitable for PD application.

## Discussion

We have successfully grown a single-crystalline ZnGa_2_O_4_ thin film on sapphire substrate by MOCVD techniques. The as-grown films exhibit the high density of oxygen vacancy defects and surface density states, which can result in deep trapping centers that increase the generation of current or trap-assisted leakage current. Consequently, the rise and decay times of as-grown PD devices are delayed, which is unsuitable for practical optoelectronic device application. However, the PD after annealing provides sufficient energy to reduce the oxygen vacancy defects and surface density states in as-grown samples to repair each other. After annealing treatment, ZnGa_2_O_4_ film shows good photoelectric characteristics. To date, no relevant research has been published yet about PD based on single-crystalline ZnGa_2_O_4_ thin films with sharp cutoff wavelength (280 nm), obviously high responsivity (86.3 A/W, under 230 nm DUV), considerably low dark current (<1 pA), short response time (<1 s), and high current on–off ratio (~10^7^). Therefore, the ZnGa_2_O_4_ PD system is a high potential candidate for solar-blind UV detection application.

## Experimental Method and Device Fabrication

ZnGa_2_O_4_ thin films were grown on c-plane (0001) sapphire substrates via a low-pressure MOCVD system. Diethyl zinc (DEZn), triethylgallium (TEGa), and oxygen (99.999%) were used as precursors. Ar (99.999%) was utilized as carrier gas to deliver DEZn and TEGa vapors to the reactor. The most excellent quality of the epilayer thin film was obtained at the low pressure of 15 Torr, growth temperature of 650 °C, and total growth time of 90 min. The flow rates of DEZn, TEGa, and oxygen were maintained at 40, 50, and 200 sccm, respectively. Previously, we have systematically investigated in details the effects of different growth parameters on the successfully grown (111)-oriented single-crystalline ZnGa_2_O_4_ films by MOCVD technique^11^. The as-grown epilayer was subsequently annealed in the furnace for 1 h at 700, 800 and 900 °C in nitrogen ambient. Crystalline quality and the epilayer orientation were measured using a high-resolution XRD (HR-XRD) system (PANalytical, X’Pert^3^ Pro MRD) with a Cu Kα line (λ = 1.541874 Å) as the radiation source. Ge (220) crystal was used as the monochromator. The surface roughness of as-grown and annealed ZnGa_2_O_4_ films was studied by atomic force microscope. The chemical bonding states and surface compositions of ZnGa_2_O_4_ films were analyzed by XPS (ULVAC-PHIPHI 5000; monochromatic Al Kα radiation, 1486.6 eV). The atomic structure of ZnGa_2_O_4_ thin films was characterized using X-ray absorption near-edge structure (XANES) and extended X-ray absorption fine structure (EXAFS) spectroscopy of Ga K-edge. Ga spectra were obtained at a beamline of 17C1 at the National Synchrotron Radiation Research Center in Hsinchu, Taiwan. The energy bandgaps (E_g_) of the ZnGa_2_O_4_ films were investigated by CL measurements. To measure the optoelectric characteristics of ZnGa_2_O_4_ films, Ti/Al/Ti/Au (50/1700/50/200 nm) electrodes with a width of 50 μm and intra-distance of 50 μm were patterned on top of the ZnGa_2_O_4_ films using photolithography. The ohmic metal was deposited by e-beam evaporation. Deuterium lamp (30 W) was used as DUV light source for photoresponse measurements. The semiconductor parameter analyzer (4155B, Keysight Technologies) was used to measure the current–voltage (I–V) characteristics of the fabricated devices in a dark ambient environment at room temperature. The schematic structure of an interdigitated finger PD with top electrodes is shown in Fig. S4 (Supplementary Information 5). In the measurement process of noise power density, the dark current was measured by precision semiconductor parameter analyzer (4156 C, Keysight Technologies) first. Then, the bias and sensitivity were set in the low noise current preamplifier (SR 570, Stanford Research Systems). Finally, the low-frequency noise from 1–1000 Hz was obtained by dynamic signal analyzer (35670 A, Keysight Technologies).

## Electronic supplementary material

Supplementary Information

## References

[1] Ozbay E (2004). High-performance solar-blind photodetectors based on Al/sub x/Ga/sub1-x/N heterostructures. IEEE J. Quantum Electron..

[2] Monroy E, Omnès F, Calle F (2003). Wide-bandgap semiconductor ultraviolet photodetectors. Semicond. Sci. Technol..

[3] Salem AA, Soliman AA, El-Haty IA (2009). Determination of nitrogen dioxide, sulfur dioxide, ozone, and ammonia in ambient air using the passive sampling method associated with ion chromatographic and potentiometric analyses. Air Qual Atmos Health..

[4] Chen H, Liu K, Hu L, Al-Ghamdi AA, Fang X (2015). New concept ultraviolet photodetectors. Materialstoday..

[5] Jung YR (2003). Pt/AlGaN metal semiconductor ultra-violet photodiodes on crack-free AlGaN layers. Jpn. J. Appl. Phys..

[6] Sang LW, Liao MY, Koide Y, Sumiya M (2011). High-temperature ultraviolet detection based on InGaN Schottky photodiodes. Appl. Phys. Lett..

[7] Feng P, Zhang JY, Li QH, Wang TH (2006). Individual β-Ga_2_O_3_ nanowires as solar-blind photodetectors. Appl. Phys. Lett..

[8] Ravadgar P (2013). Effects of crystallinity and point defects on optoelectronic applications of β-Ga_2_O_3_ epilayers. Optics Express..

[9] Li C, Bando Y, Liao M, Koide Y, Golberg D (2010). Visible-blind deep-ultraviolet Schottky photodetector with a photocurrent gain based on individual Zn_2_GeO_4_ nanowire. Appl. Phys. Lett..

[10] Shen YS, Wang WK, Horng RH (2017). Characterizations of Metal-Oxide-Semiconductor Field-Effect Transistors of ZnGaO Grown on Sapphire Substrate. IEEE J. Of the Elec. Devices Soc..

[11] Horng R-H, Huang CY, Ou S–L, Juang T-K, Liu P–L (2017). Epitaxial Growth of ZnGa_2_O_4_: A New, Deep Ultraviolet Semiconductor Candidate. Cryst. Growth Des..

[12] Alema F (2017). Solar blind photodetector based on epitaxial zinc doped Ga2O3 thin film. Phys. Status Solidi A..

[13] Omata T, Ueda N, Ueda K, Kawazoea H (1994). New ultraviolet-transport electroconductive oxide, ZnGa_2_0_4_ spinel. Appl. Phys. Lett..

[14] Han S (2011). Photoconductive gain in solar-blind ultraviolet photodetector based on Mg_0.52_Zn_0.48_O thin film. Appl. Phys. Lett..

[15] Huang CY, Horng RH, Wu DS, Tu LW, Kao HS (2013). Thermal annealing effect on material characterizations of β−Ga_2_O_3_ epilayer grown by metal organic chemical vapor deposition. Appl. Phys. Lett..

[16] Guo DY (2015). Unipolar resistive switching behavior of amorphous gallium oxide thin films for nonvolatile memory applications. Appl. Phys. Lett..

[17] Kim JH (2011). Correlation of the change in transfer characteristics with the interfacial trap densities of amorphous In–Ga–Zn–O thin film transistors under light illumination. Appl. Phys. Lett..

[18] Guo DY (2014). Oxygen vacancy tuned Ohmic-Schottky conversion for enhanced performance in β-Ga_2_O_3_ solar-blind ultraviolet photodetectors. Appl. Phys. Lett..

[19] Wang Z (2016). Tuning the selectivity toward CO evolution in the photocatalytic conversion of CO_2_ with H_2_O through the modification of Ag-loaded Ga_2_O_3_ with a ZnGa_2_O_4_ layer. Catalysis Science & Technology..

[20] Behrens P, Kosslick H, Tuan VA, Fröba M, Neissendorfer F (1995). X-ray absorption spectroscopic study on the structure and crystallization of Ga-containing MFI-type zeolites. Microporous Materials..

[21] Criado GM (2014). Crossed Ga_2_O_3_/SnO_2_ Multiwire Architecture: A Local Structure Study with Nanometer Resolution. Nano letters..

[22] Li YB (2010). Efficient assembly of bridged β-Ga_2_O_3_ nanowires for solar-blind photodetection. Adv. Funct. Mater..

[23] Singh K, Rawal I, Punia R, Dhar R (2017). X-ray photoelectron spectroscopy investigations of band offsets in Ga0.02Zn0.98O/ZnO heterojunction for UV photodetectors. J. Of Appl. Phys..

[24] Lou Z, Li L, Shen G (2015). High-performance rigid and flexible ultraviolet photodetectors with single-crystalline ZnGa_2_O_4_ nanowires. Nano. Research..

[25] Zheng Q (2012). High-Responsivity Solar-Blind Photodetector Based on Mg_0.46_Zn_0.54_O Thin Film. IEEE Elec. Dev. Lett..

[26] Feng P, Zhang JY, Wan Q, Wang TH (2007). Photocurrent characteristics of individual ZnGa_2_O_4_ nanowires. J. of Appl. Phys..

[27] Tebano A, Fabbri E, Pergolesi D, Balestrino G, Traversa E (2012). Room-Temperature Giant Persistent Photoconductivity in SrTiO_3_/LaAlO_3_ Heterostructures. Acs Nano..

[28] Chuanga MY (2014). Density-controlled and seedless growth of laterally bridged ZnO nanorod for UV photodetector applications. Sensors and Actuators B..

[29] Chang SJ (2008). GaN-Based Schottky Barrier Photodetectors With a 12-Pair MgxNy–GaN Buffer Layer. IEEE J. of Quantum Electron..

[30] Feng H (2013). Fabrication and UV-sensing properties of one-dimensional β-Ga_2_O_3_ nanomaterials. Phys. Status Solidi A..

[31] Zhang M (2013). NaTaO3-Based Ultraviolet Photodetector With Capacitive Efficacy. IEEE Elec. Dev. Lett..

[32] Zhao Y (2009). Ultraviolet Photodetector Based on a MgZnO Film Grown by Radio-Frequency Magnetron Sputtering. ACS Applied Mater. & Interface..

[33] Zhang M (2013). Ultraviolet Detector Based on SrZr0.1Ti0.9O3 Film. IEEE Elec. Dev. Lett..

[34] Zhang M (2013). High response solar-blind ultraviolet photodetector based on Zr_0.5_Ti_0.5_O_2_ film. Appl.Surf. Sci..

[35] Xing J, Changchun Z, Erjia G, Yang F (2012). High-Performance Ultraviolet Photodetector Based on Polycrystalline SrTiO_3_ Thin Film. IEEE Sens. J..

[36] Li L (2010). Ultrahigh-performance solar-blind photodetectors based on individual single-crystalline In2Ge2O7 nanobelts. Adv. Mater..

