# Supplementary material for: Deep-Ultraviolet Photodetectors Based on Epitaxial ZnGa2O4 Thin Films
Source: Sci Rep. 2018 Sep 19;8:14056. doi: 10.1038/s41598-018-32412-3 (PMC6145910; doi:10.1038/s41598-018-32412-3)
Supplement: Supplementary file 1 — Supplementary Information [file 41598_2018_32412_MOESM1_ESM.pdf]

Supplementary Information

Deep-Ultraviolet Photodetectors Based on Epitaxial $ZnGa_2O_4$ Thin Film

Si-Han Tsai[1], Sarbani Basu[1], Chiung-Yi Huang[2], Liang-Ching Hsu[3], Yan-Gu Lin[3], Ray-Hua Horng[1*]

[1]Institute of Electronics, National Chiao Tung University, Hsinchu, 300, Taiwan(R.O.C.)
[2]Institute of Precision Engineering, National Chung Hsing University, Taichung, 402, Taiwan(R.O.C.)
[3] National Synchrotron Radiation Research Center (NSRRC), Hsinchu, Taiwan(R.O.C)
*E-mail: rhh@nctu.edu.tw

Supplementary Information

To evaluate the effect of thermal annealing on the energy level distribution, as-grown and annealed $ZnGa_2O_4$ epilayers were measured by Cathodoluminescence (CL) and shown in Fig. S1. It was found that the epilayers with and without annealing showed not obvious band to band (~5.1 eV) emission. The strong intensity of as-grown $ZnGa_2O_4$ on the CL spectra is found around 335 nm (3.7 eV) and the UV emission was attributed to recombination of self-trapped excitons [1]. The UV emission in $ZnGa_2O_4$ may result from interactions between (Zn_{Ga})' and (V_O, V_{Ga}) defects, which appears to have a quenching" effect on the activities of (V_O, V_{Ga}), reducing the intrinsic blue emission while at the same time promoting the UV emission [2]. After annealing treatment, the broad emission band in UV region (280~400 nm) totally dropped. It indicated that the defects resulting the UV emission reduced in annealing process. To analysis the band edge emission, the intensity curve in deep UV region (200~280 nm) was magnified. Theoretically, the Eg of ZnGa2O4 is about 5.2-5.4 eV. The weak emission bands located at 242 nm (E_g ~ 5.25 eV) and 230 nm (5.4 eV) are observed in the as-grown and annealed $ZnGa_2O_4$ epilayers, respectively, which has been shown in the inset of Fig. S1. The weak emission bands could be attributed to the band-to-band emissions for these two epilayers. Nevertheless, the annealed $ZnGa_2O_4$ thin film showed lower CL peak intensity as compare to as-grown thin films. It could be due to the defect density of as-grown films being reduced through annealing process.

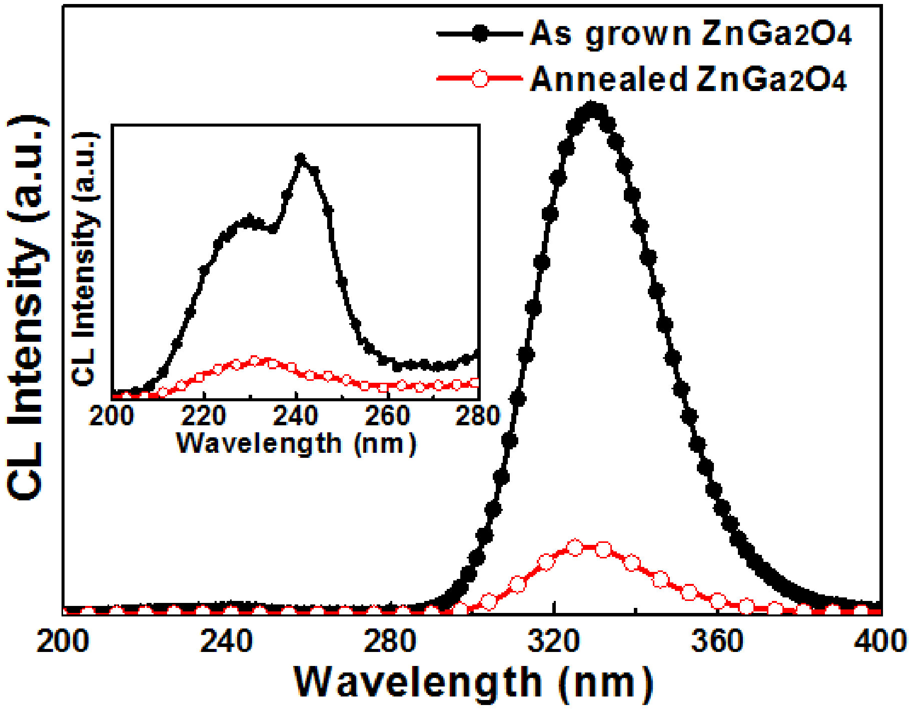

Figure S1. CL spectra of as-grown and annealed at 800°C $ZnGa_2O_4$ thin films.

Fig. S2 (Supplementary information 2) shows the dark current (I_{dark}) and UV-illuminated photocurrent (I_{light}) at 230 nm I–V curves for MSM DUV PDs fabricated using as-grown and annealed (700, 800, 900°C) $ZnGa_2O_4$ epitaxial films. Obviously, the dark current can be reduced with increasing annealing temperature. The devices made of the 700°C annealed films have an ratio of $I_{light}/I_{dark} \sim 10^6$ orders of magnitude, which further increases to 10^8 and then decreases to 10^4 for annealed 800°C and 900°C devices, respectively. Comparative responsivity results between as-grown and annealed devices revealed that lowering of responsivity with annealing temperature is good well with the observed reduction in photocurrent as supported in Fig. S2.

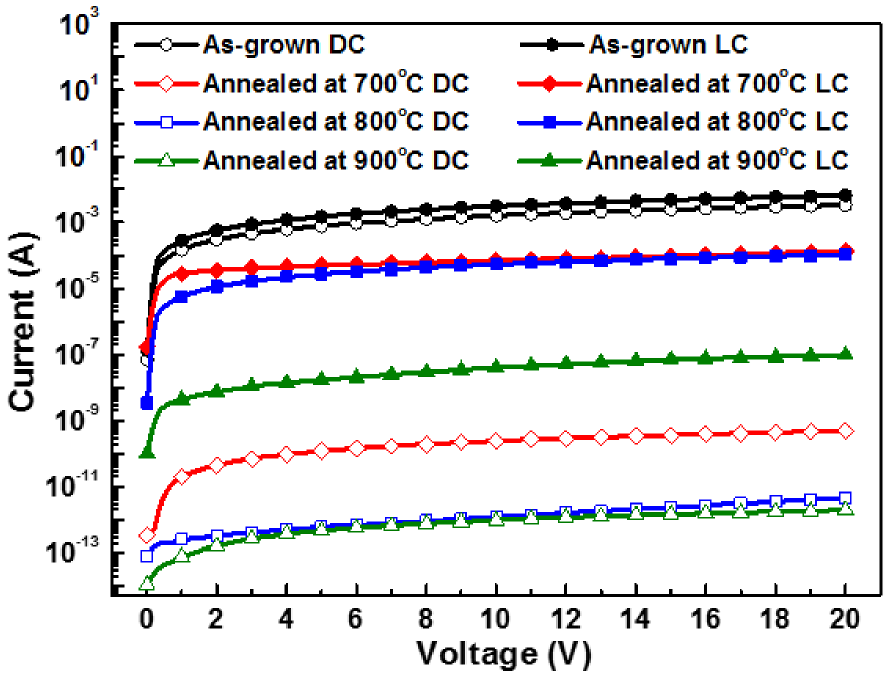

Figure S2. Dark current (DC) curve and photocurrent (LC) at 230 nm curve for as-grown (black) and annealed in 700°C (red), 800°C (blue) and 900°C (green) $ZnGa_2O_4$ UPDs.

Fig. S3 (Supplementary information 3) shows the complete photoresponse of as-grown and annealed (800°C) $ZnGa_2O_4$ DUV PDs. Obviously, there exists the PPC effect in the as-grown $ZnGa_2O_4$ PDs. It was results from the deep level traps. The deep level traps could be demonstrated by CL and shown in Fig. S1. In the as-grown $ZnGa_2O_4$, the deep level traps could come from the oxygen vacancy and surface density states. On the contrary, the rise and decay time are about 0.5 and 0.7 s, respectively, for the annealed (800°C) $ZnGa_2O_4$ DUV PDs. The PPC effect can be alleviated in the annealed (800°C) $ZnGa_2O_4$ DUV PDs.

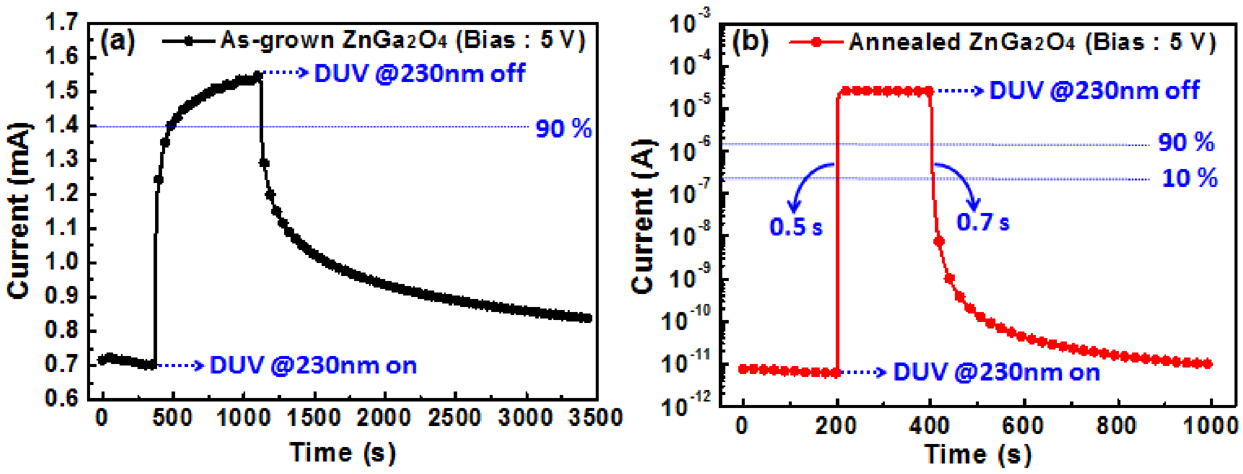

Figure S3. Complete response of (a) as-grown and (b) annealed $ZnGa_2O_4$ UPDs

Table 1. Characteristics of the different ternary metal oxides of ultraviolet photodetector.

Metal oxide	Device structure	Dark current (A)	Light of detection (nm)	Rejection ratio	Responsivity (AW^{-1})	Response time (T_r, T_f)	Ref
Zn_2GeO_4 NW (MOCVD)	MSM		254	DUV to vis: 10^4	38.3 A/W, 8 V	Tr ~12 s<br>T_f ~ 0.6 s	9
$Ga_{0.02}Zn_{0.98}O$ (PLD)	MSM		370		22.62%	Tr ~ 44 s<br>T_f ~ 91 s	23
Zn_2GeO_4 NW (VLS)	MSM	0.4 nA	350	On/off ratio: 120	3174 A/W	Tr ~ 15 s<br>T_f ~ 10 s	24
β-Ga_2O_3:Zn	MSM	23 nA	232	5×10^4	210 A/W	Tr ~ 3.2 s<br>T_f ~ 1.4 s	12
$ZnGa_2O_4$ (MOCVD)	MSM	8.5 pA	254			Tr ~ 2 s<br>T_f < 1 s	26
β-Ga_2O_3 NW (MOCVD)	MSM	0.01 nA	254	UV to dark current: 42		Tr ~ 0.32 s<br>T_f ~ 0.08 s	30
$NaTaO_3$ (Hydrothermal)	MSM	1.5 nA, −5 V	290	UV to dark current: 13		Tr ~ 4.6 s<br>T_f ~ 15.4 s	31
$Mg_{0.47}Zn_{0.53}O$ (RF sputtering)	MSM	3 pA, 5 V	260	UV to vis: > 4	10.5 mA/W, 5 V	Tr ~ 10 ns<br>T_f ~ 30 ns	32
$SrZn_{0.1}Ti_{0.9}O_3$ (Sol–gel)	MSM	41 pA, 5V	250	UV to vis: 43	94 mA/W	Tr ~ 3.8 ms<br>T_f ~ 565 ms	33
$Zr_{0.5}Ti_{0.5}O_2$ (Chemical solution)	MSM	17 pA	310	UV to dark current: 3	620 mA/W	Tr ~ 424.1 s<br>T_f ~ 154 ms	34
$SrTiO_3$ (Sputtering)	MSM	0.4 nA, 50 V	200–250	UV to vis: 2	105 mA/W	Tr ~ 330 ps<br>T_f ~ 480 ps	35
$In_2Ge_2O_7$ (Sol-gel)	p–n		335	UV to dark current: 600	3.9×10^5 A/W	T_f ~ 3 ms	36
$ZnGa_2O_4$ (MOCVD)	MSM	1 pA, 5 V	233	UV to dark current: 10^7	86.3 A/W, 5 V	Tr ~ 0.5 s<br>T_f ~ 0.7 s	(This work)

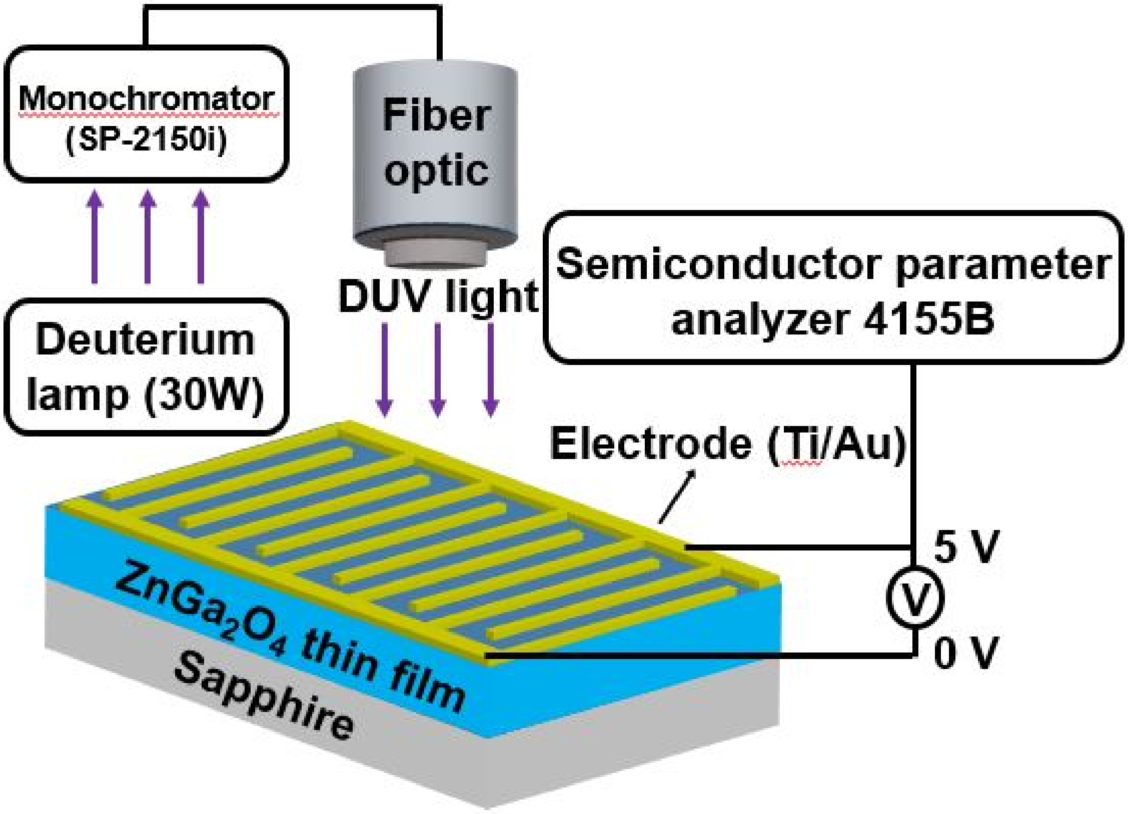

Figure S5. Metal–semiconductor–metal (MSM) structure for $ZnGa_2O_4$ thin-film DUV PDs.
